# Environmental Footprints in Food Services: A Scoping Review

**DOI:** 10.3390/nu16132106

**Published:** 2024-07-02

**Authors:** Nathalia Sernizon Guimarães, Marcela Gomes Reis, Bruna Vieira de Lima Costa, Renata Puppin Zandonadi, Conrado Carrascosa, Edite Teixeira-Lemos, Cristina A. Costa, Hmidan A. Alturki, António Raposo

**Affiliations:** 1 Department of Nutrition, Nursing School, Universidade Federal de Minas Gerais, Alfredo Balena Avenue, 190, Room 314, Santa Efigênia, Belo Horizonte 30130-100, Minas Gerais, Brazil; reis.marcelanutri@gmail.com (M.G.R.); brunavlcosta@gmail.com (B.V.d.L.C.); 2 Department of Nutrition, School of Health Sciences, University of Brasilia (UnB), Campus Darcy Ribeiro, Asa Norte, Brasilia 70910-900, Brazil; renatapz@unb.br; 3 Department of Animal Pathology and Production, Bromatology and Food Technology, Faculty of Veterinary, Universidad de Las Palmas de Gran Canaria, Trasmontaña s/n, 35413 Arucas, Spain; conrado.carrascosa@ulpgc.es; 4 CERNAS Research Centre, Polytechnic University of Viseu, 3504-510 Viseu, Portugal; etlemos3@gmail.com (E.T.-L.); amarocosta@esav.ipv.pt (C.A.C.); 5 King Abdulaziz City for Science & Technology, Wellness and Preventive Medicine Institute—Health Sector, Riyadh 11442, Saudi Arabia; halturki@kacst.edu.sa; 6 CBIOS (Research Center for Biosciences and Health Technologies), Universidade Lusófona de Humanidades e Tecnologias, Campo Grande 376, 1749-024 Lisboa, Portugal

**Keywords:** environmental, epidemiology, food services, food waste, sustainability

## Abstract

The collective meals market generates significant revenue for the world economy. Food services are responsible for consuming large amounts of water and energy, as well as generating a substantial volume of waste, which is often improperly disposed of. Given the unchecked expansion of food services, the lack of proper management of environmental resources can undermine sustainability principles, posing a threat to future generations. This scoping review aimed to synthesize the existing scientific literature on carbon and water footprints in food services, describing the main methods and tools used and what strategies have been proposed to mitigate the high values of these footprints. The search for articles was performed on 6 June 2024 in seven electronic databases, using MeSH Terms and adaptations for each database from database inception. The search for local studies was complemented by a manual search in the list of references of the studies selected to compose this review. It included quantitative studies assessing footprints (water or carbon) in food services and excluded reviews, studies that reported footprints for diets, and protocols. A total of 2642 studies were identified, and among these, 29 were selected for this review. According to the findings, it was observed that meats, especially beef, contribute more to water and carbon footprint compared to other proteins. Mitigation strategies for the water footprint include promoting plant-based diets, menu changes, and awareness.

## 1. Introduction

The global economy heavily relies on the collective food market. The food market is expected to generate USD 10.07 trillion in revenue by 2024. The market is expected to grow at 6.53% annually until 2028 [1]. On the one hand, there is the growth of a market that generates profits and jobs, but on the other, if this growth is not managed properly, it may not fit into the concept of sustainability, compromising future generations [2,3,4,5]. The UN’s sustainable development goals are responsible consumption and production [6,7].

According to the Brundtland report [8], sustainable development satisfies current needs without compromising future generations’ ability to meet their own needs. This report highlights five dimensions of sustainable development: social (focusing on poverty reduction and social organization), economic (maintaining ecosystem productivity), ecological (preserving natural resources as the foundation of biodiversity), spatial (focusing on a balanced rural–urban configuration), and cultural (regarding local community specificities, identities, and traditions). Sustainability refers to the balance between economic development and natural resource preservation, ensuring economic growth and better living conditions for current and future generations. More than just promoting sustainability in collective food production, the goal is to avoid compromising future generations [9].

Food services use a lot of energy and water and produce a lot of waste, much of which is not disposed of properly [10]. By utilizing management and control tools in the meal production process—such as keeping track of technical preparation sheets, implementing selective collection, and using a checklist linked to good environmental practices—as well as providing training to staff members and raising user awareness of the need to reduce waste, nutritionists in food services can reduce their negative environmental effects. Many working there seldom pay attention to environmental burden, or most of them have no tools to estimate the water and carbon footprint created by preparation and cooking. The pressing need to combat climate change and advance sustainable practices has led to a notable increase in awareness of the environmental effects of the food service industry in recent decades [11].

Carbon footprint measurement focuses on the amount of greenhouse gas emissions associated with food production, processing, transportation, and preparation. The water footprint measures the total volume of water consumed in the food supply chain. The complexity and diversity of food services, including restaurants, cafeterias, and institutions like schools and hospitals, pose significant challenges in understanding and mitigating their environmental impacts [12]. A synthesis of the evidence of carbon and water footprints in food is crucial for guiding nutritionists to better environmental practices and sustainable policies.

About this context, this review aims to provide insights for nutritionists who plan menus in food services, institutional kitchens, or restaurants interested in promoting sustainable practices. In this context, we have therefore structured this review to answer three questions:

1. What are the most frequent tools used for evaluating carbon and water footprints in food services?
2. What strategies have been proposed and implemented to mitigate these challenges?
3. Which knowledge gaps remain and which areas require more attention in future research?

## 2. Materials and Methods

This study was written as a scoping review according to JBI tools. This study was registered on the Open Science Framework platform (https://osf.io/9xuys/, accessed on 28 June 2024).

### 2.1. Data Source and Search Strategy

The search for information was conducted using the electronic databases of PubMed, Embase, LILACS, IBECS, BINACIS (via the Virtual Health Library), Web of Science, and Scopus, as well as gray literature. The review was conducted until June 2024.

The list of terms identified in MeSH (medical subject headings), Emtree, or DeCS (health sciences descriptors) used to search for articles was as follows: “Carbon Footprint”, “Water”, “Water Resources”, “Environmental Indicators”, “Environment”, ”Meals”, “Menu Planning”, and “Food Services”. The information search strategy included combining the descriptors and using Boolean indicators “OR” and “AND.” The correspondence between Portuguese, Spanish, and French was also used. Furthermore, a manual search was performed on all the included study reference lists to identify potential local studies (Table S1).

### 2.2. Outcomes

The primary outcomes were the carbon footprint and water footprint. The carbon footprint measurement focuses on the amount of greenhouse gas emissions associated with food production, processing, transportation, and preparation.

The water footprint measures the total volume of water consumed in the food supply chain.

### 2.3. Eligibility Criteria

Quantitative studies that described footprints (water or carbon) in food services were included. The data collection of these studies was performed using the menus of food services. Abstracts presented at congresses were also included. We excluded narrative, integrative, scoping, rapid, or systematic reviews; studies that reported footprints only for diets and not for service foods; and protocols. We did not include studies that tested the applicability of new menus or studies that only covered food preparations, specific foods, or food groups, i.e., we only included menus (breakfast, morning snack, lunch, afternoon snack, dinner, or supper). Also, studies that exclusively estimated carbon dioxide amount, which is just a part of greenhouse gas emissions, were excluded. No restrictions were imposed on the dates or places of publication.

### 2.4. Study Selection and Data Extraction

We uploaded the electronic search results from the defined databases to the Rayyan Qatar Computing Research Institute app for systematic reviews. Two reviewers independently screened titles and abstracts. These reviewers independently assessed each eligible study to determine whether they met the inclusion criteria. A third independent reviewer addressed any discrepancies. 

The research team prepared and applied a data extraction spreadsheet to summarize the following data from the studies: reference (name and year of publication of the study), study location (country), title, journal, objectives, study period (weeks), food service studied, instruments or references used to calculate the water and/or carbon footprints, main results, and final considerations with pointing out the gaps in the area.

## 3. Results

**Figure 1:**
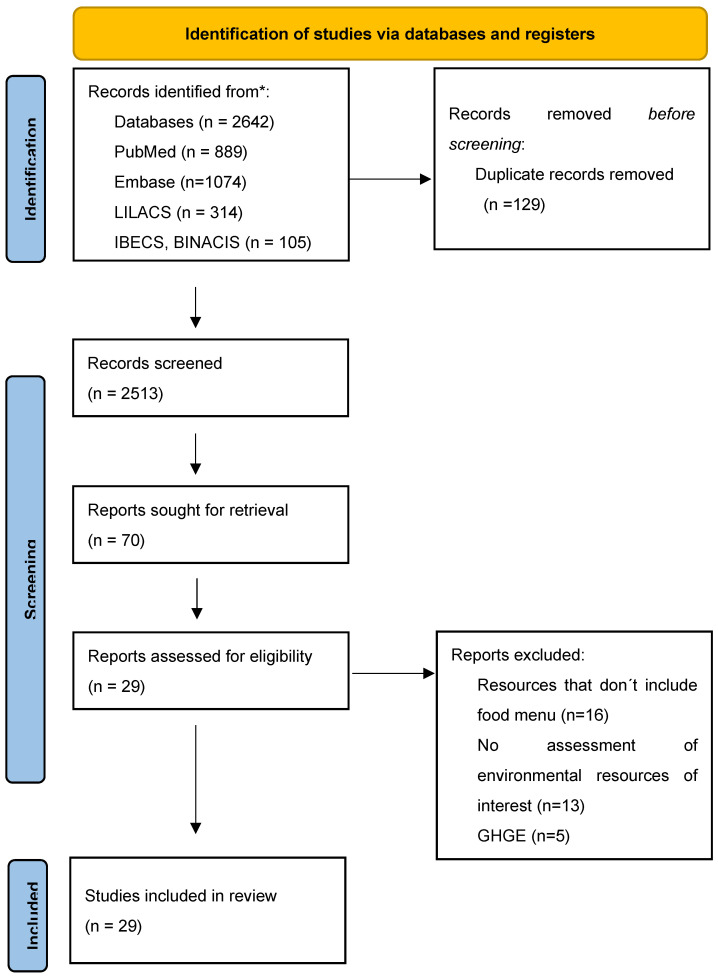
Flowchart for the selection of studies, 2024. * Consider, if feasible to do so, reporting the number of records identified from each database or register searched (rather than the total number across all databases/registers).

A total of 2642 studies were found via electronic database. After excluding 129 duplicates, 2513 titles and abstracts were examined. Of these, 70 records were evaluated by the full text and 41 were excluded according to the eligibility criteria, as described in Table S2. After text eligibility was assessed, 29 studies were selected for this review (Figure 1).

For didactic purposes, we will present the topic divided into three categories: studies aimed at quantifying water footprint (*n* = 9), studies aimed at quantifying carbon footprint on menus (*n* = 11), and both (*n* = 9) [Table 1]. Most (*n* = 14) studies evaluated the environmental waste at university restaurants. Three searches evaluated footprint at hospitals, two at restaurants, one at a hotel, and eight at school canteens. One did not present the food service observed.

### 3.1. Water Footprint (WF)

Of the studies that evaluated WF (*n* = 18), most (*n* = 11) were conducted in Brazil, three in Spain, two in Turkey, one in England, and one in Uruguay. Most studies were conducted and published in the last three years. According to most studies, the monitoring duration ranged from one week to seven years [Table 2].

Four studies evaluated school canteens, ten evaluated menus at university restaurants, and two evaluated hospital restaurants. The WF was evaluated using various parameters, including the following:

1. «Huella hídrica» (HH) in Spanish, or Water footprint in English [37];
2. Use of the “The Value of Food” calculator at www.elvalordelsaliments.cat/calculadora/ accessed on 20 May 2024 [42,43];
3. Preparation sheets and water footprint estimates were obtained for each food item, including surface or subterranean water (blue), rainwater (green), and water required to balance the system’s pollution load [12];
4. Water–Energy–Food (WEF) Nexus Framework (FAO, 2014) [44].

Huella hídrica was used for Strasburg et al., 2023 [37]. The “The Value of Food” calculator was used by Laurentiis, Hunt, and Rogers, 2017 [24]; Aytekin-Sahin et al., 2023 [16]; Manera et al.2023 [25]; Garcia et al., 2020 [20]; and Saleki et al., 2023 [33]. The food items were used by Alencar Lima et al., 2021 [13]; Alves Lima et al., 2023 [15]; Falco et al., 2021 [18]; Paião, 2021 [31]; and Silva, 2023 [34]. The Water–Energy–Food (WEF) Nexus Framework was used by Verão, 2018 [38]; Mesquita and Carvalho, 2023 [29]; and Laurentiis, Hunt, and Rogers, 2017 [24].

**Table 2:** Water footprints: results for each individual study of the review.

Reference	Instruments or References	Main Results	Strategies to Mitigate Footprints
Alencar Lima et al., 2021 [13]	Surface or underground water (blue water), rainwater (green water), and water necessary to assimilate the system’s pollution load [12]	The average WF of omnivorous foods was 2423.55 L, while that of vegetarians was 506.44 L; that is, the former had a footprint almost five times larger than the latter. Beef had the greatest impact on WF. On vegetarian menus, a difference on WF was seen between the offering of eggs and textured soy protein, showing that eggs have a greater impact.	NI
Alves Lima et al., 2023 [15]	Surface or underground water (blue water), rainwater (green water), and water necessary to assimilate the system’s pollution load [12]	Omnivorous menus with beef have larger footprints compared to other types of meat. For vegetarian menus, the footprints were larger in meals that contained eggs. The use of rice as the base of the main dish on the vegetarian menu had a larger footprint than most menus with textured soy protein. Among the omnivorous menus, the WF averages were higher when using beef and among the vegetarian menus. There were no differences between the use of textured soy protein and other legumes/ cereals with regard to WF. The average WF of the omnivorous menus was 2423.55 L, while that of the vegetarian menus was 506.44 L.	Changes in eating behavior, reducing red meat consumption. It is suggested to encourage the consumption of omnivorous menus with less meat in general, but mainly red meat, and to offer more attractive and diverse vegetarian menus.
Aytekin-Sahin et al., 2023 [16]	WF was calculated in L/kg per product for each food using the WF factors [42,43].	The mean water footprint of all menus was higher than that of the Mediterranean diet. Compared to other hospitals, Hospital D served larger portions and more frequent meals with red meat, while Hospital A, which had the lowest carbon and water footprints, served chicken and vegetables more frequently. The differences in water footprint were attributed to the varying frequencies of red meat, chicken, and vegetables. Animal-based foods, especially beef and lamb, significantly increase the water footprint of menus. Instead of removing red meat entirely, reducing its amount, substituting it with chicken, fish, or legumes, and incorporating more vegetables and fruits can be more easily adopted by patients.	There is a need for tools to evaluate menus objectively and quickly. In addition to providing adequate and balanced food services in institutions such as hospitals, schools, and nursing homes, it is essential to consider the water footprint of these menus. Compliance with the Mediterranean diet in food service institutions is important not only for supporting human health but also for reducing the environmental impact of the food system. Recommendations to reduce the environmental footprint of food systems must be balanced with dietary requirements for health. Therefore, patient satisfaction should also be taken into consideration when revising menus to be more sustainable. By balancing sustainability with patient satisfaction, menus can serve as a tool to improve public health, with hospital food services focusing on menus suitable for the Mediterranean diet.
Falco et al., 2021 [18]	Surface or underground water (blue water), rainwater (green water), and water necessary to assimilate the system’s pollution load [12]	The menus presented the following values for the analyzed WF of 3343 (449–6470) L. The entries presented, on average, 63.7 L of WF. The average for the main dishes was 2030.7 L for WF. The average for garnishes was 102.1 L WF; for side dishes, it was 219 L WF. The average for vegetarian preparations was 410.2 L of WF. Beef-based preparations were those with the highest footprints (Lisboeta beef and sugo meatballs), with a negative environmental impact; therefore, their supply should be avoided.	Include footprint values when nutritionists plan menus.
Garcia et al., 2020 [20]	Water–Energy–Food (WEF) Nexus Framework [43]	Menus that include main dishes with beef presented the highest WF. Menus that include other products of animal origin, such as yogurt and cold cuts, showed worse WF profiles when considering an energy-based functional unit (100 kcal). The result shows the lowest energy consumption (457 kcal) at a high WF score. Menus relying more on animal products, specifically meat and dairy products, imply higher WF scores. Beef as a main dish corresponds to 48% and 66% of the total WF. Dairy products contribute to a total 25% of total WF. Menus rich in dairy products, including milk, butter, and cheese, in the preparation of the dish (vegetable cannelloni); yogurt in the dessert; and cheese in the snack (cheese sandwich) form a category representing 62% of the total WF. Vegetables and fruits represent between 3% and 28% of the total WF. Meals rich in plant-based foods imply lower WF scores.	Promoting reduction in animal food by increasing the consumption of plant-based products Reducing beef consumption by incorporating alternatives such as chicken or pork. Combining beef on menus with food products with a better environmental profile and high nutritional quality (such as fruit and cereals). Attention should be paid to assumptions and data quality (e.g., waste production factors and energy use related to cooking). Modify menus with higher scores, introducing alternative food combinations always following the Atlantic diet recommendations.
Garcia et al., 2021 [21]	Water–Energy–Food (WEF) Nexus Framework [43]	Products that account for the largest share of consumptive WFs are of animal origin: they represent 59–86% of the total water demand in all menus. When beef is included at lunch, the highest consumption WF scores are identified and differ significantly from those on other menus. Beef/beef products (meat and dairy) are by far the main focus regarding WF. Vegetables and fruits represent between 2% and 14% of total contributions to WF. Lunch is the main meal responsible for WF demand, with contributions ranging from 38% to 95%.	Incorporating turkey, pork, and chicken meat as an alternative to beef for lunch. Combining beef with more environmentally friendly food products (e.g., fruits or milk) on menus.
Hatjiathanassiadou et al., 2019 [22]	Per capita value of each food item was obtained from prep sheets and water footprint estimates [45] for vegetal foods and [46] for animal foods.	Comparing the results of the traditional and vegetarian menus, it can be seen that the per capita value of WF was significantly higher (*p* < 0.0001) in the traditional menu.	The results of the research offer subsidies so that the managers of food services can better plan actions related to the organization of the menus offered, aiming at reducing the environmental impact of meal production.
Kilian et al., 2021 [23]	Per capita value of each food item was obtained from prep sheets and water footprint estimates [45].	Concerning the WF assessment, the averages for the omnivorous menus are significantly higher than those for the vegetarian menu. The lowest WF refers to days when dishes were based on chicken, pork, or fish. The lowest WF refers to days when dishes were based on textured soy protein and roasted vegetables. In the vegetarian profile, the highest WF is related to the days in which the menu established the association of two types of legumes. When evaluating the WF values of foods, it was found that vegetables have lower WF when compared to products of animal origin. WF was proportionally lower whenever foods of animal origin were reduced, especially red meat.	NI
Laurentiis, Hunt, and Rogers, 2017 [24]	Water use was measured using the water footprint (WF) concept, calculated as the total volume of direct and indirect water used, consumed, and polluted. The values of WF associated with the production of the food items analyzed were extracted from two databases [42,43].	Vegetarian dishes were responsible for 40% of the total impact of WF. This was mainly due to chocolate desserts, which alone were responsible for 19% of the total WF.	Therefore, a strategy to reduce the water footprint (WF) could be replacing chocolate desserts with other types of desserts, including healthier fruit options. Additionally, implementing strategies to minimize plate waste, such as serving flexible portion sizes, improving the quality of meals to make them more appealing, and developing educational projects for schools, caterers, and children, could ensure that the resources involved in meal production (and the associated emissions) are not wasted.
Manera et al., 2023 [25]	“The Value of Food” calculator [42,43]	From 301.97 to 178.88 l H_2_O (−40.76%) in water footprint	To reduce the amount and frequency of animal protein foods in school menus
Nogueira et al., 2020 [30]	Per capita value of each food item was obtained from prep sheets and water footprint estimates [45] for vegetal foods and [46] for animal foods.	WF per capita average found for the six restaurants evaluated in the present study was 2165.8 L of water per lunch meal. Restaurants 1 to 4 had WF values above 2100.0 L/kg of meal/day. This may be because these restaurants offer a high per capita serving of animal protein.	Decrease in beef and increase in the supply of fish and proteins of vegetable origin, such as beans, lentils, peas, and chickpeas, would be the alternative for reducing the water footprint without compromising the nutritional quality of the menus offered. However, it is known that budget limitations and cost increases are factors to be considered in Brazilian public institutions.
Paião, 2021 [31]	Surface or underground water (blue water), rainwater (green water), and water necessary to assimilate the system’s pollution load [12]	The omnivorous menu had an average usage of 2423.55 L, while the vegetarian menu had a lower average WF, with only 506.44 L. Animal products are the main determinants of higher WF. When comparing the types of meat offered in the omnivorous protein dish, beef presented greater relevance when compared to other meats used (pork, chicken, and fish). A vegetarian diet has a smaller effect on WF, indicating that this diet can reduce the use of water resources used by the food production system.	Decrease in the consumption of red meat and increase the consumption of proteins of plant origin such as legumes, nuts, fruits, vegetables and legumes. Reducing or avoiding the supply of red meat, both in frequency and quantity (portion). The use of indicators such as water footprints in institutional restaurants also supports educational actions and public policies so that they prioritize the consumption of foods with a lower environmental impact.
Saleki et al., 2023 [33]	Determine the use of water throughout the production and supply chains of products. The water footprint factors obtained from the literature review were used to calculate the water footprints of the nutrients, animal products, and agricultural products in the menus. To calculate the water footprint, the food gram amounts of each menu were multiplied by their own water footprint, and finally, the total water footprint of the menu was calculated [42,43].	The results of our study determine that the wide variety of dishes on the menus and the frequent use of animal-based dishes in the menus are factors that increase the water footprint. The water footprint value of the sustainable menu examples based on plant-based nutrition with less meat are lower than the current menus. In addition, they are balanced and sufficient in terms of micro-micronutrients.	Based on the modelling performed in this study, routine meal menus with sustainable meal menus would be more economical and more nutritious, in addition to having positive environmental impacts. Therefore, it is recommended to plan for the implementation of such changes in line with green and sustainable university and economic goals in countries. Furthermore, an alternative vegetarian menu could have positive effects on health and environmental sustainability.
Silva, 2023 [34]	Surface or underground water (blue water), rainwater (green water), and water necessary to assimilate the system’s pollution load [12]	An average WF value of 57.97 L/day/per capita was identified in the menus. The highest water footprint index was identified in the breakfast that offered bread with egg and mango juice, presenting a value of 60,845.95 L/meal, followed by the fruit salad, which presented 15,166.32 L/meal.	NI
Strasburg et al., 2015 [35]	Per capita value of each food item was obtained from prep sheets and water footprint estimates [45] for vegetal foods and [46] for animal foods.	Plant-origin products (cereals, legumes, vegetables, and fruits) were responsible for 22.1% of the total water footprint (WF). Products of animal origin represented 77.9% of the WF, with beef cuts accounting for the highest percentage (62.2% of the total in this group). The average WF per menu preparation was 2099 L per day. But on days when the main dish was beef, the daily average rose to 2717 L, and on the days when it was chicken, the daily average was 1172 L per day. There was a 44.2% reduction in WF in relation to the general daily media per menu.	Actions in planning menus
Strasburg et al., 2016 [36]	Per capita value of each food item was obtained from prep sheets and water footprint estimates [45] for vegetal foods and [46] for animal foods.	The relationship between the impact of WF and kcal results in a value of more than two liters of WF for each kcal. Analysis of this relationship between food groups reveals values ≥70% for food groups of animal origin. The Pearson correlation between the variables kg of animal products and WF demonstrates a very strong positive correlation (0.704), indicating that the greater the quantity in kg of inputs of animal origin, the higher the WF value. A very strong positive correlation (0.666) was also observed between kg of plant products and WF, indicating that the greater the quantity in kg of plant inputs, the higher the WF value.	Actions in planning menus and the consumption of inputs that will be used and how much they can impact the environment directly or indirectly. Products of animal origin have greater impacts on the environmental (WF) and financial contexts. Plant-based foods provided a better energy supply but generated greater waste generation.
Strasburg et al., 2023 [37]	Water footprint [42,43]	A vegetarian diet has a smaller effect on WF.	Less animal protein; seasonal consumption can mitigate the impact of weather on food supply in school cafeterias
Verão, 2018 [38]	Per capita value of each food item was obtained from prep sheets and water footprint estimates [44]	The products necessary for the production of meals were considered and classified according to their common characteristics, establishing three different groups: (1) Agricultural and Derivatives; (2) Products of Animal Origin; and (3) Disposables. Among the products with the highest values for WF, those of animal origin stand out, such as beef shoulder cuts, chuck meat, ducklings, and soft thighs, representing around 49% of the WF in the supply chain. Another product with higher values for WF was paper towels, with 2.95% of the total value, being a product not directly associated with food.	General orientations: Control and monitoring of water consumption; through the installation of sectoral water meters; Replacement of old hydraulic systems; Preparation of Effluent Reuse Projects; Development of rainwater harvesting projects; Preparation of feasibility projects for the use of ozone for disinfection of hospital beds and in laundry processes. Animal products, agricultural products, and derivatives: Reduction in wasted consumption in the cafeteria; Hire suppliers with certification of good production practices.

NI = Not informed.

#### 3.1.1. Water Footprint (WF) in Food Services: Key Findings

The presence of animal-derived foods, particularly beef and dairy products, is linked to significantly higher WF levels in the analyzed menus, according to 14 studies. Menus featuring beef as the main dish have the highest WF scores, making them the primary contributors to water consumption. Furthermore, the presence of beef is consistently identified as the main cause of increased hydration compared to other animal protein sources, such as pork and fish. This suggests that reducing or avoiding beef consumption can significantly impact reducing hydration (Table 2).

Studies show that vegetarian menus have significantly lower hydration levels compared to meat-based menus, especially when based on vegetable proteins such as soy protein and eggs. Vegetarian recipes that include eggs as a protein source have a higher hydration level than soy protein preparations. Studies highlight the importance of considering not only the nutritional value of foods, but also their environmental impact, particularly in terms of water demand (Table 2).

#### 3.1.2. Strategies for Reducing High Water Demand in Food Services

According to ten studies, the primary strategies for reducing high hydration levels in menus include reducing the consumption of animal foods, particularly red meat, and increasing the consumption of plant proteins such as fruits, vegetables, and legumes. This can be accomplished by developing menus that prioritize appealing and diverse vegetarian options, as well as introducing meat alternatives such as chicken, pork, and turkey in place of beef.

In addition, strategies such as water consumption control and monitoring, replacing old hydraulic systems, reusing effluents, and collecting rainwater can help reduce water consumption in food services. Using certified suppliers with good production practices is a key measure to reduce waste and improve resource efficiency.

Analytical tools such as life cycle costing highlighted the importance of meal preparation and identified food waste, particularly vegetables, as a critical issue. Sensitivity analysis revealed that modifying the composition and preparation of meals can significantly reduce environmental impact and costs, highlighting the importance of considering food waste when seeking sustainable solutions in food services (Table 2).

### 3.2. Carbon Footprint (CF)

Six of the studies that evaluated CF were conducted in Brazil, four in Spain, one in Italy, one in the United States, two in Turkey, one in Finland, one in England, one in China, and three in Portugal. Seven studies evaluated school canteens, seven evaluated university restaurants, two evaluated restaurants, two evaluated hospitals, and one evaluated a hotel. One did not provide information (Table 3).

**Table 3:** Carbon footprints: results for each individual study of the review.

Reference	Instruments or References	Main Results	Strategies to Mitigate Footprints
Alencar Lima et al., 2021 [13]	Data from the study by Garzillo et al. (2019) [12] were used to calculate the footprints.	There is a significant difference between omnivorous and vegetarian menus, where the former have an average of six times higher GHG emissions (2320.06 gCO_2_eq) than vegetarian menus (402.57 gCO_2_eq). There is also a significant difference between the protein dishes on vegetarian menus. Preparations with eggs had a higher CF than those containing vegetables, legumes, and cereals.	NI
Almeida, 2021 [14]	CF of the menus was carried out using the environmental database by Garzillo et al. (2019) [12]. For foods that did not have CF values available in the literature, foods were chosen with values from the same group or with the same ingredients.	Higher per capita and total CF values were found in menus with higher consumption of red meat and number of diners. CF values also showed an increase in weeks when more preparations with red meat were offered, compared to weeks with chicken, pork, or fish, which had a lower CF. The results may also be related to the supply frequency and the per capita value of preparations served.	Offer preparations with other types of animal protein (fish, chicken) without the need to remove red meat from menus
Alves Lima et al., 2023 [15]	Data from the study by Garzillo and collaborators (2019) [12] were used as a reference to calculate the footprints, considering that each product may present variations depending on specific regional characteristics, such as soil and climate. When calculating each preparation, the WF and CF values of the cooked foods were considered.	Omnivorous menus with beef have larger footprints compared to other types of meat. For vegetarian menus, the footprints were larger in meals that contained eggs. The use of rice as the base of the main dish on the vegetarian menu had larger footprints than most menus with textured soy protein. Among the omnivorous menus, the WF averages were higher when using beef and among the vegetarian menus. There were no differences between the use of textured soy protein and other legumes/legumes/cereals with regard to WF. The average WF of the omnivorous menus was 2423.55 L, while that of the vegetarian menus was 506.44 L.	Reduction in the consumption of red meat in order to encourage the consumption of omnivorous menus with less meat in general, but mainly reducing red meat and offering more attractive and diversified vegetarian menus.
Aytekin-Sahin et al., 2023 [16]	The carbon emission of each food per kg product using the CF factors [42,43].	The average carbon footprint of the menus was higher than that of the Mediterranean diet, except in one hospital. This exception had the lowest carbon footprint and served chicken and vegetables more frequently. However, there was a hospital that served larger portions and more frequent meals with red meat compared to the other hospitals. These differences in carbon footprint were primarily due to the varying frequencies of red meat, chicken, and vegetables. Animal-based foods, especially beef and lamb, significantly contribute to the carbon footprint of menus. Instead of eliminating red meat entirely, reducing its amount and replacing it with protein sources such as chicken, fish, or legumes, along with adding more vegetables and fruits, may be initial steps that patients can adopt more easily.	There is a need for tools to evaluate menus objectively and quickly. In addition to providing adequate and balanced food services in institutions such as hospitals, schools, and nursing homes, it is essential to consider the carbon footprint of these menus. Compliance with the Mediterranean diet in food service institutions is important not only for supporting human health but also for reducing the environmental impact of the food system. Recommendations to reduce the environmental footprint of food systems must be balanced with dietary requirements for health. Therefore, patient satisfaction should also be taken into consideration when revising menus to be more sustainable. By balancing sustainability with patient satisfaction, menus can serve as a tool to improve public health, with hospital food services focusing on menus suitable for the Mediterranean diet.
Costa et al., 2023 [17]	To calculate the menu’s carbon footprint, the TUCO Greenhouse Gas (TUCO) calculator was used (TUCO, 2021).	Three menus with the lowest protein food portion adequacy, with a high portion of animal protein sources, were also the menus with the highest environmental impact (more CO_2_ emissions). On the other hand, the other menus presented lower values of CO_2_ emission, since they contained components of cereals and vegetables and more adequate amounts of protein foods. It was verified that the menu with the highest carbon footprint had the lowest nutritional adequacy and that, in turn, had the lowest adequacy in terms of food portions. In contrast, the menu with the greatest nutritional and portion adequacy also contributed less GHG emissions.	It is relevant to consider the reformulation of restaurant menus and meals, considering the following: (1) the way they are described and presented to the client, valuing the vegetable component of the menu (vegetables, cereals, legumes) in detriment of the animal component, (2) reducing food portions and providing adequate information about meals so that people can make informed and healthy choices; (3) improving skill levels of employees; (4) creating facilities for leftovers. All these initiatives in restaurants can lead to a reduction in average daily energy consumption, reducing the environmental impact of carbon footprint and reducing food waste.
Falco et al., 2021 [18]	Data from the study by Garzillo et al. (2019) [12] were used to calculate the footprints.	The menus presented the following values for the analyzed footprints 3388 (689–7201) gCO_2_eq for CF. The entries presented, on average, 57.6 gCO_2_eq of CF. The average for the main dishes was 2258.9 gCO_2_eq for CF. The average for garnishes was 80.4 gCO_2_eq; for side dishes, it was 100.9 gCO_2_eq. The average for vegetarian preparations was 519.2 gCO_2_eq. Beef-based preparations had the highest footprints (Lisboeta beef and sugo meatballs) and had a negative environmental impact.	Better menu planning is needed to create a menu with less environmental impact.
Franchini et al., 2023 [19]	Each customizable item’s carbon footprint (g CO_2_ equivalent) was determined following the methodology described in Malan et al. The Carbon Footprint Scorecard developed by UCLA based on estimates of GHG (greenhouse gas) emissions was used. Based on the Planetary Health Diet recommendations provided by the Eat–Lancet Commission, a daily value (DV) of the dietary carbon footprint was calculated and the food categories were ranked according to their contribution to it: low (0–25% of the DV), medium (26–50% of the DV), and high (>50% of the DV).	The most chosen food categories were cheese (27% and 25%), pork (18% and 17%), and poultry (15% and 14%) for products with high to medium carbon footprint (66% and 58%) and fruit (11% and 11%) and vegetables (15% and 17%) for foods with low carbon footprint (37% and 42%). The placing of lower-carbon-impact options at the top of the menu resulted in higher sales than when they were placed at the bottom of the menu. The purchase of higher-carbon-impact items, such as pork, poultry, and cheese, decreased when they were located at the end of the list. The beef sales were not impacted by the menu reordering and remained stable even when listed at the end of the menu.	Educational and campaign materials such as university programs, seminars, Teaching Kitchen events, posters, and infographics with information about the carbon footprint of food in the dining halls, as well as high- and low-carbon-footprint icons added to online and in-person menus.
Garcia et al., 2020 [20]	CF was conducted considering the entire food supply chain until the food is ready for consumption. Therefore, the stages of food production, retailing, and cooking in the nursery kitchen were considered in the analysis.	The food production stage is the main stage responsible for GHG emissions, representing around 94% of total CF. Kitchen activities in electrical appliances are responsible for about 4% of total CF. The GHG emissions of the omnivorous menus analyzed range from 0.84 to 3.09 kg CO_2_eq per menu supplied to each child. The variations in the CFs are linked to the type of foodstuffs. Menus rich in animal foods are associated with higher GHG emission rates and CF. Menus that include beef have the highest CF scores (2.00 and 3.09 kg CO_2_eq per menu). Menus that include cold meat-based sandwiches also have a significant CF score, where the production of this type of meat plays a key role because of background processes involved (≈11.3 kg CO_2_eq/kg cold meat). The group based on milk and dairy products is also an important source of GHG emissions since, in all cases, cow milk-based products have been considered. Therefore, menus incorporating cheese have been associated with higher CF scores even without meat in the composition. Fish and seafood are responsible for the largest contribution to GHG emissions from the group of non-dairy protein sources. Vegetables, fruit, cereals, and pulses report very low contributions to the CF scores. Fish menus that do not incorporate any meat source and include chicken breast are those with the lowest CF scores.	Introducing alternative food combinations with food products with a better environmental profile and high nutritional quality (such as fruit and cereals), always following the Atlantic diet recommendations.
Garcia et al., 2021 [21]	CF emerges as the environmental indicator of GHG emissions produced during the product’s life cycle, reporting a final index in CO_2_ equivalents. An LCA approach has been followed to determine the GHG emissions associated with each menu served at the canteen. Thus, three life cycle stages have been considered: foodstuffs production, distribution, and cooking.	Non-dairy sources of protein are by far the main group responsible for GHG emissions in all menus, where this position is occupied by milk and dairy products (in fact, this menu incorporated four dairy products in the form of milk, butter, cheese, and yogurt). Non-dairy protein sources include not only meat but also fish, seafood, eggs, and pulses. The effect of meat on GHG emissions is outstanding. Lunch is the meal with the greatest effect on CF scores. In this sense, lunch implies contributions ranging from 66% to 86%. Menus that include beef meat in the second course have the highest effect on the overall CF score. In terms of kg CO_2_eq per meal, menus with cold meat-based sandwiches, yogurt, and fresh cheese sandwiches have the highest CF index associated with the afternoon snack (around 0.51 kg CO_2_eq·afternoon snack), while the menu serving cereals with milk and apple as an afternoon snack was associated with the lowest CF index (0.31 kg CO_2_eq). Milk is the dairy product with the lowest CF score (1.39 kg CO_2_eq).	Replacing beef with other meats that have less impact, such as turkey, pork, and chicken, as well as combining beef with more environmentally friendly food products (e.g., fruit or milk) on menus.
Laurentiis, Hunt, and Rogers, 2017 [24]	Indicator of carbon footprint (CF), measured in kg CO_2_ equivalent, which quantifies how much heat each greenhouse gas (CO_2_, N_2_O, and CH_4_) traps in the atmosphere when compared to the amount of heat trapped by CO_2_ for a time horizon of 100 years. The CF from cradle to delivery to school kitchens was calculated by adding two components: the CF of production and the CF of transport. The former component was obtained through a review of LCA literature studies where the CF of 1 kg of each food item calculated from cradle to farm gate (or factory gate for processed items) was extracted. The later component was calculated separately based on the country of production and on the transport mode [44].	In particular, beef- and lamb-based dishes contributed significantly to the total CF (being responsible for 38% of the total emissions).	If a number of strategies to minimize plate waste were put in place, such as serving flexible portion sizes, improving the quality of meals to make them more appealing, and developing educational projects for schools, caterers, and children alike, the resources involved in the production of those meals (and the associated emissions) would not be used (and released) in vain. Additionally, a reduction in meat dishes would reduce the environmental impact of the service. This could be achieved by introducing more attractive vegetarian alternatives to traditional meat-based dishes, partially replacing the meat content of dishes with plant-based protein sources (such as pulses), and substituting red meat dishes with white meat and fish options.
Manera et al., 2023 [25]	Using the “The Value of Food” calculator—www.elvalordelsaliments.cat/calculadora/ ENT Foundation and The Waste Agency of the Catalan Government (accessed on 28 June 2024) and based on examples of school menu plannings for children aged 6 to 12 in the 2012 and 2020 guide comparison, the carbon footprint (kg of CO_2_), the water footprint (L of H_2_O), and the land use (m_2_) were calculated. The calculations were made based on the amounts of main protein foods (pulses, meat, fish, eggs) on the 2012 menus versus the 2020 menus. Dairy products and nuts were excluded due to their low presence in school menus.	From 2012 to 2020, the carbon footprint in menu planning showed a reduction from 12.55 to 5.64 kg of CO_2_ (−55.06%)	Reduction in the amount and frequency of animal protein foods in school menus.
Martinez et al., 2020 [26]	To quantify the CF, the different processes involved along the supply chain are converted into GHG emissions using carbon factors. These factors are relative to carbon dioxide and expressed in kg CO_2_ equivalent per reference unit [45].	Regarding the overall carbon footprint (CF) of the baseline menu and the six alternative menus, the baseline menu has the highest CF at 24.39 kg CO_2_ eq/person/monthly lunch meal, followed by the menu without fish. The menu without fish shows only a 4% reduction compared to the baseline menu, primarily because fish dishes are substituted with meat dishes. However, there is still a reduction in the CF of the menu without fish due to the substitution of fish dishes with eggs, which have a lower CF than some types of fish, such as hake (70% lower) and salmon (9% lower). On the other hand, the astringent menu exhibits the lowest total CF at 14.77 kg CO_2_ eq/person/monthly lunch meal, followed by the menu without meat. The reductions in CF for both the astringent menu and the menu without meat, which are 40% and 30% lower than the baseline menu, respectively, are attributed to the elimination of beef dishes. The astringent menu has a lower CF than the menu without meat due to its restrictive calorie intake, which is 13% lower.	A series of recommendations have been presented to improve the sustainability of food consumption. In particular, policy makers are encouraged to consider environmental aspects in school dietary guidelines and national dietary guidelines in general. It is believed that incorporating knowledge of the environmental performance of foods into dietary guidelines could potentially promote more sustainable eating habits. In light of these findings, a possible optimization of the alternative menus could involve using plant-based protein sources as substitutes for meat, fish, and eggs.
Matzembacher et al., 2020 [27]	A life cycle assessment (LCA) was performed to understand the environmental impacts of this wasted food. The goal of the LCA was to estimate the carbon footprint of the food waste generated in different restaurant configurations to identify the hotspots in the system. That is, the researchers wished to understand which food waste fractions contribute significantly to the carbon footprint and to support the design of effective measures to reduce the global warming potential associated with the wastage of food.	NI	NI
Melo et al., 2023 [28]	To calculate the footprint of the meals under analysis, a BARILLA DATABASE for double pyramid 2015 was used [46].	The presence of disposable spoons on yogurt days only represents 2.8% of the carbon footprint. The only meal without animal products is the one with the lowest carbon footprint, and the biggest is when two products of animal origin are present. In this way, intermediated school meals with a higher number of dairy products have a greater carbon footprint than others.	Given the results, promoting intermediate meals that align with the Mediterranean eating pattern, including the inclusion of fruits and vegetables, will enhance the nutritional and environmental adequacy of these meals. This approach not only promotes health and well-being but also serves as a vehicle for nutrition education and encourages healthy food consumption in schools. Although historically well-framed, school food supply programs do not emphasize the importance they can have in providing food for children from economically disadvantaged families enough, thereby contributing significantly to food security.
Mesquita and Carvalho, 2023 [29]	To ensure consistency in the use of these studies and relevance in the quantitative estimation of the carbon footprint of meals and foods, the LCA components were reviewed, and the results were adjusted according to the food items’ estimated “Degree of supply of the internal market”. To best ensure relevance, accuracy, and consistency in the results, the functional unit was considered [44,47].	By analyzing food consumption in Portugal through the common meals that are usually eaten, it is shown that this food consumption includes high carbon footprint foods, such as beef, sheep, crustaceans, and cheese. On the other hand, vegetables and fruits, which have low carbon footprints, are less frequent in diets in Portugal. The results reveal that most vegetarian meals eaten in Portugal have a lower carbon footprint than non-vegetarian meals eaten in Portugal. In addition, if animal protein was replaced by plant-based protein in Portugal’s meals, its carbon footprint would be substantially smaller.	It should be noted that it is possible to consume mainly non-vegetarian meals with a lower carbon footprint than solely vegetarian meals if the main protein sources are low carbon footprint animal foods, such as eggs and horse mackerel. In plant-based meals, using tofu and soy-based products ensures the bioavailability of essential amino acids. Additionally, the “daily consumption of foods with complementary essential amino acid (EAA) profiles” (e.g., combining beans and rice) is recommended as an alternative.
Pulkkinen et al., 2016 [32]	A simplified carbon footprint assessment was conducted based on researchers’ previous LCA studies, literature reviews, and the new scientific literature.	According to the results, the carbon footprints of main courses and side salads varied significantly. On average, a main course with a side dish accounted for 45% of the meal’s carbon footprint, while a side salad contributed almost 30%, milk 20%, and bread less than 10%. Vegetarian meals generally showed significantly lower emissions compared to average meals. Dishes like vegetable soups, curries, tofu, beans, and lentil dishes had the lowest emissions. On the other hand, creamy soups and lasagna with cheese had emissions around the average of all meals. The highest emissions among vegetarian dishes were observed in Greek and goat cheese salads, which included cheese and vegetables grown in greenhouses during winter. Fish main courses generally had low impacts, except for some salmon dishes. Whether a fish-based meal fell below or above the average depended largely on the emissions from the accompanying side salad. Meat dishes typically had emissions at or above the average level. Moderate consumption of meat as part of a well-balanced meal can help keep the overall environmental impact at the average level.	By composing a meal differently, or even just changing the recipe of the main dish or side salad, climate change impact could be decreased significantly. To enable simple and reliable carbon footprinting of meals, a transparent database of climate change impacts of food needs to be created and integrated with restaurants’ current IT systems. Carbon footprinting allows design of versatile meals, including small amounts of ingredients of animal origin, which are more attractive than vegan meals for many consumers. Development of a sufficiently reliable database of LCA results needs further research and should be given adequate attention before restaurant purchases are guided by the information.
Saleki et al., 2023 [33]	To calculate the footprint of the meals under analysis, a BARILLA DATABASE for double pyramid 2015 was used [46].	According to the findings, both the December and February menus in the refectory had higher carbon footprint values compared to the menus designed as sustainable examples. The study identified that the wide variety of dishes and frequent inclusion of animal-based dishes are factors contributing to the increased carbon footprint in the current menus. In contrast, the carbon footprint of sustainable menu examples based on plant-based nutrition with reduced meat content was lower than that of the current menus. Additionally, these sustainable menus were found to be balanced and sufficient in terms of micronutrient content.	Based on the modelling performed in this study, routine meal menus with sustainable meal menus would be more economical and more nutritious, in addition to having positive environmental impacts. Therefore, it is recommended to plan for the implementation of such changes in line with green and sustainable university and economic goals in countries. Furthermore, an alternative vegetarian menu could have positive effects on health and environmental sustainability.
Vidal et al., 2015 [39]	The carbon footprint can be quantified at a product level, as in the life cycle assessment (LCA) described by the International Organization for Standardization.	Special attention needs to be paid to the carbon footprint of red meat as it has a very significant effect. The average carbon footprint of these two countries is therefore much higher than the normal diet in the Spanish hospital analysed.	The use of Mediterranean diet, characterized by its abundance of plant foods and moderate consumption of red meat, has a lower environmental impact compared to the average US diet. Moreover, it aligns closely with the public health recommendations issued by the World Health Organization. Therefore, promoting the Mediterranean diet can not only support environmental sustainability but also contribute to better public health outcomes.
Volanti et al., 2022 [40]	LCA methodology was applied to determine the carbon footprint (CF) of each meal, and the resulting value was then related to the food’s energy content. The CF (kg CO_2_ eq.) estimates the total amount of GHGs directly and indirectly emitted during the production, while the food energy, expressed in kJ, is the energy released within the body when food nutrients (carbohydrates, fats and proteins) are burned. The identification of these two parameters allowed us to calculate the Carbon Footprint/Food Energy (CFE) index, defined as the ratio between the CF (g CO_2_ eq./dish) and the food energy (kJ/dish). CFs associated with each specific ingredient were mainly collected from the international EPD system and Ecoinvent 3.5 database. For nutritional value, the CREA website was taken as a reference.	Between the dairy products, cheese and butter presented a higher CF, while in the case of milk and eggs, it was demonstrated to be lower. In general, fresh fruit, vegetables, cereals, and legumes had the lowest CO_2_ eq. contribution, although dry fruit should be considered the best option because it combines a low CF with high food energy (low CFE index). The first courses resulted in the best combination of the two parameters because many of them had a high energy content that justified the environmental impact. Side dishes had an even smaller CF, but their caloric contribution to the person was also lower, resulting in a CFE index similar to that of first courses. On the contrary, second courses generally had high CF value but intermediate food energy, which makes them the most discouraged choice from an environmental/nutritional point of view.	Changes to the school feeding policy, consequently reducing the environmental impact.
Wu et al., 2019 [41]	Carbon footprint (CF) refers to direct and indirect greenhouse gas emissions to the environment related to wasted food per kilogram over its life cycle, measured in kilogram carbon dioxide equivalent units (kgCO_2_eq). This was calculated as follows: a variable represents carbon related to waste during agricultural production; a variable represents carbon related to waste during processing (mainly primary processing of grain and reprocessing of bean products); a variable represents carbon related to waste during food transportation to Beijing; a variable represents carbon related to waste during preparation and consumption (mainly cooking energy); and a variable represents carbon related to waste during disposal (including final transportation).	Staple foods dominated the source compositions of the CF (39%). The CF of staple foods was mainly derived from agricultural production (23.1%) and canteen consumption (58.6%). Although the share of vegetables in food waste reached 42%, their contribution to CF was 34%, mainly deriving from canteen consumption (68.8%). In contrast, although meat accounted for only 9% of food waste, its contribution to CF was higher. The previously determined food waste of 73.7 g/cap/meal resulted in about 258.6 gCO_2_ eq CF. Considering the total population of 1.3 million university students in Beijing, accounting for 5.9% of the city’s resident population, student-related food waste would thus cause 227.6 tCO_2_ eq CF. According to our questionnaire survey results, students ate out roughly twice per week.	Should focus on improving taste and quality by hiring more proficient chefs and improving ingredient quality. Provide more appropriate food amounts by offering diverse serving portions. Adjusting the management system with little financial cost can provide more appropriate food portions by changing the price and size of each dish. Can improve ingredient quality.

NI = Not informed.

The studies examined carbon footprints in health services using various methodologies. In general, methods such as the following were used:

1. The Carbon Footprint Scorecard [6] developed by UCLA based on estimates of GHG (greenhouse gas) emissions. A daily value (DV) of the dietary carbon footprint was calculated and the food categories were ranked according to their contribution to it: low (0–25% of the DV), medium (26–50% of the DV), and high (>50% of the DV)—Franchini et al., 2023 [19].
2. Considering the entire food supply chain until the food is ready for consumption. Therefore, the stages of food production, retailing, and cooking in the nursery kitchen were considered in the analysis—Garcia et al., 2020 [20].
3. The life cycle assessment (LCA) approach was followed to determine the GHG emissions associated with each menu served at the canteen. Thus, three life cycle stages were considered: foodstuff production, distribution, and cooking—Garcia et al., 2021 [21]; Lauretti et al., 2017 [24]; Matzembacher et al., 2020 [27]; Mesquita and Carvalho, 2023 [29]; Pulkkinen et al., 2016 [32]; Vidal et al., 2015 [39]; and Volanti et al., 2022 [40].
4. “The Value of Food”—www.elvalordelsaliments.cat/calculadora/—(ENT Foundation and The Waste Agency of the Catalan Government)—(assessed on 28 June 2024) The “The Value of Food” calculator was used by Laurentiis, Hunt, and Rogers, 2017 [24]; Aytekin-Sahin et al., 2023 [16]; Manera et al., 2023 [25]; Garcia et al., 2020 [20]; and Saleki et al., 2023 [33].

#### 3.2.1. Carbon Footprint in Food Services: Key Findings

Studies on carbon footprint in food services have revealed significant trends and patterns. Regarding food choices, products with high or medium carbon footprints, such as cheese, meat, and poultry, were the most popular. In contrast, low-impact foods, like fruits and vegetables, received less preference. Reorganization strategies, such as putting low-impact options at the top of the list, led to increased consumption of these items and decreased sales of high-impact products like beef, poultry, and cheese. Food production is the leading source of greenhouse gas emissions (GHGE), accounting for 94% of the total carbon footprint.

Meat, particularly beef, was identified as the primary contributor to increased carbon footprint in the menus studied. Lactose-based products, particularly cheese and milk, had a significant impact. Plant-based foods, like vegetables, cereals, and legumes, had relatively low carbon footprints.

The studies also show a significant decrease in carbon footprint from 2012 to 2020. Furthermore, significant differences in carbon footprints were found between carnivorous and vegetarian menus, with the former emitting six times more GHGE than the latter. In terms of food waste management practices, it was discovered that basic foods were responsible for the majority of emissions of nitrogen, phosphorus, carbon, while fruits represented a significant portion of food waste.

#### 3.2.2. Strategies for Reducing High Carbon Footprint in Food Services

The studies examined various strategies to reduce carbon footprints in food services, focusing on reducing animal product consumption and promoting sustainable practices. Some of the key strategies proposed include the following:

1. Cardboard reorganization: placing environmentally friendly options at the top of the list increased sales and reduced consumption of high-carbon products.
2. Promoting plant-based diets: reducing consumption of animal products, particularly beef, in favor of plant-based options has been recommended as a way to reduce the carbon footprint associated with eating. Replacing beef with alternatives like chicken, pork, and plant-based products can significantly reduce greenhouse gas emissions.
3. Improving food quality through strategies such as hiring experienced chefs and improving ingredient quality can reduce food waste and improve resource efficiency.
4. Implementing sustainable food policies in educational institutions and university restaurants, such as offering more diverse vegetarian menus and reducing food waste, can significantly reduce carbon footprint.

In summary, the proposed strategies aim to encourage changes in eating habits and promote sustainable practices in food services, with the goal of reducing greenhouse gas emissions and mitigating the environmental impact of food production.

## 4. Discussion

The environmental impact was assessed by evaluating studies quantifying the water footprint (WF) and carbon footprint (CF) of the foods and menus. University restaurants (14 studies) were evaluated in the largest number of studies when compared to school canteens (8 studies) and hospital restaurants (3 studies) or restaurants (2 studies). The results found for all the footprints evaluated form a consensus that menus that included beef or animal products in general, including eggs, dairy, and dairy products, had a greater impact. In addition, foods of plant origin, such as fruit, vegetables, cereals, and legumes, as well as vegetarian menus as a whole, had a low environmental impact. This cannot be seen in omnivorous menus, which had a high environmental impact.

Mitigating environmental impacts through combined food production and consumption can help address critical points and overcome sustainability challenges. Nutritionists play a key role in menu planning and shaping institutional policies that use impact indicators and tools, particularly concerning carbon and water footprints. This allows for identifying key products with the greatest impact on sustainability and leads to better outcomes [27]. Furthermore, using these indicators predicts direct and indirect effects on health of the general population, such as promoting health and conserving natural resources through lower water and carbon footprints. As a result, it provides the ability to manage educational campaigns and public policies to achieve better sustainability outcomes.

Strategies for motivating consumers through educational and interactive campaigns have been implemented to increase awareness of sustainability and promote improvements in food choices in light of the size of the impact generated by foods. However, the most relevant actions in studies [13,19,35] are those that promote the substitution of high-impact foods (red meat products) with low-impact foods (vegetable products) in the menus. This action does not depend solely on dietary choices, but also on meal planning [18,20,23]. The relationship between educational campaigns, interventions, and menu planning is critical for promoting sustainable food choices. Our findings suggest that nutritionists can significantly reduce the environmental impact of institutional food services by implementing educational and interactive campaigns. These initiatives not only raise awareness about the environmental footprint of different foods but also encourage the substitution of high-impact animal products with low-impact plant-based alternatives [48]. The studies reviewed highlight the effectiveness of these interventions in various settings. For example, meal planning and educational efforts in schools have led to substantial reductions in carbon and water footprints. These outcomes underscore the importance of integrating education and campaigns into the toolkit of nutritionists for sustainable menu planning [23].

Some studies evaluated meals planning and intervention in food services considering the sustainability impact [45,46,47]. A case study conducted in Spain planned meals for schoolchildren for lunch (20-day menus) and promoted changes in 47 dishes regarding nutritional, environmental, and economic parameters. The results show CF reductions from 13 to 24% and reductions in budget from 10 to 15%, maintaining the nutritional aspects of the original menus. The authors, using the optimization model, showed the importance of avoiding the dishes with a high CF and cost, which are low in iron content but high in protein and cholesterol [49]. A study conducted in Italy, also with a schoolchildren canteen, used a systematic procedure to change menus to have a lower environmental impact [49]. The procedure optimally used some recipes over three courses of twenty-day menu lunches. The menus proposed by the model were considered environmentally friendly compared to the previous menus. It was possible to reduce more than 20% of GHGE, more than 40% of CO2eq, and more than 20% of water consumption [50]. A study in Croatia aimed to optimize sustainability in nine schools by replacing animal foods with plant-based foods. The results show that new meals with a low CF (<376 g CO2 eq) provide all macronutrients and energy needs [50].

This review presents limitations. As a limitation, we observed the lack of representation of all countries worldwide in the evaluated studies. This may impact the generalization of the findings. However, this was a close representation of a clinically diverse setting from several continents. Another important factor to consider is the amount of time spent in the study periods of the evaluated studies, which may not be enough to detect acceptance or stimulation from the media over time [14]. Future research should be encouraged to ensure a robust body of literature, sustainability indicators for each environmental impact, and support for nutritionists and businesses through the assessment of foods, beverages, and menus, as well as resources and instruments that aid in the daily practice of reducing environmental impact.

Despite its limitations, we performed a comprehensive literature search that included various independent databases. Search, selection, and data extraction applied to the selected studies were performed separately and in duplicate by two researchers, and a third party was accessed to resolve disagreements. As strengths, we pointed out the updating of the researched information, since most studies were conducted and published in the last three years.

## 5. Conclusions

This review approached current knowledge on carbon and water footprints in food services and identified research trends. Studies evaluated water footprint using mainly Huella hídrica in Spanish and “The Value of Food” calculator methods. Carbon footprints were mainly analyzed using the Carbon Footprint Scorecard, considering the entire food supply chain until the food is ready for consumption; the life cycle assessment (LCA); and “The Value of Food” calculator. The results show that animal-derived foods on menus were linked to the highest WF and CF levels, while vegetarian menus have significantly lower WF and CF levels.

The review highlighted significant findings on the environmental impact of food services, particularly focusing on the carbon and water footprints (CF and WF) of various menus. The consensus across the evaluated studies is clear: menus featuring animal-derived foods exhibit the highest WF and CF levels, while vegetarian menus have notably lower levels.

The strategies to reduce WF and CF in food services must focus on lowering animal product offerings and promoting sustainable practices. Some of the key strategies proposed include cardboard reorganization, placing environmentally friendly options at the top of the list to increase sales and reduce consumption of less sustainable products; promoting plant-based diets; improving food quality to reduce food waste and improve resource efficiency; promoting consumer awareness of the environmental impact of food; and implementing sustainable food policies in educational institutions and university restaurants, such as offering more diverse vegetarian menus and reducing food waste. These can significantly reduce carbon footprint. Studies highlight the importance of considering not only the nutritional value of menus, but also their environmental impact.

## Figures and Tables

**Table 1:** Characteristics of included studies.

Reference	Study Location	Study Period	Food Service	Number of Food Services Evaluated
Alencar Lima et al., 2021 [13]	Brazil	4 weeks	University restaurant	01
Almeida, 2021 [14]	Brazil	61 weeks	University restaurant	06
Alves Lima et al., 2023 [15]	Brazil	NI	University restaurant	01
Aytekin-Sahin et al., 2023 [16]	Turkey	4 weeks	Hospital restaurant	05
Costa et al., 2023 [17]	Portugal	1 week	Hotel restaurant	01
Falco et al., 2021 [18]	Brazil	NI	University restaurant	01
Franchini et al., 2023 [19]	USA	13 weeks	University restaurant	01
Garcia et al., 2020 [20]	Spain	26 weeks	School canteens	01
Garcia et al., 2021 [21]	Spain	26 weeks	School canteens	01
Hatjiathanassiadou et al., 2019 [22]	Brazil	8 weeks	University restaurant	01
Kilian et al., 2021 [23]	Brazil	NI	University restaurant	02
Laurentiis, Hunt and Rogers, 2017 [24]	England	2 weeks	School canteens	01
Manera et al., 2023 [25]	Spain	417 weeks	School canteens	92
Martinez et al., 2020 [26]	Spain	NI	School canteens	01
Matzembacher et al., 2020 [27]	Brazil	NI	Restaurants	06
Melo et al., 2023 [28]	Portugal	NI	School canteens	Not specific. “schools of a municipality in the central region of Portugal”
Mesquita and Carvalho, 2023 [29]	Portugal	NI	NI	Not specific.
Nogueira et al., 2020 [30]	Brazil	57 weeks	University restaurant	06
Paião, 2021 [31]	Brazil	4 weeks	University restaurant	01
Pulkkinen et al., 2016 [32]	Finland	NI	Restaurants	03
Saleki et al., 2023 [33]	Turkey	3 weeks	University restaurant	01
Silva, 2023 [34]	Brazil	NI	School canteens	01
Strasburg et al., 2015 [35]	Brazil	2 weeks	University restaurant	01
Strasburg et al., 2016 [36]	Brazil	52 weeks	University restaurant	05
Strasburg et al., 2023 [37]	Uruguay	52 weeks	University restaurant	01
Verão, 2018 [38]	Brazil	NI	Hospital restaurant	01
Vidal et al., 2015 [39]	Spain	1 week	Hospital restaurant	01
Volanti et al., 2022 [40]	Italy	NI	School canteens	01
Wu et al., 2019 [41]	China	17 weeks	University restaurant	06

NI = Not informed.

## Data Availability

No new data were created or analyzed in this study.

## Supplementary Materials

The following supporting information can be downloaded at https://www.mdpi.com/article/10.3390/nu16132106/s1. Table S1: Indexers used to select publications; Table S2: Full-text excluded articles and reasons. References [51,52,53,54,55,56,57,58,59,60,61,62,63,64,65,66,67,68,69,70,71,72,73,74,75,76,77,78,79,80,81,82,83,84,85,86,87,88,89] are cited in the Supplementary Materials.
