# Supplementary material for: Environmental Footprints in Food Services: A Scoping Review
Source: Nutrients. 2024 Jul 2;16(13):2106. doi: 10.3390/nu16132106 (PMC11243183; doi:10.3390/nu16132106)
Supplement: Supplementary file 1 [file nutrients-16-02106-s001.zip › nutrients-3087319-supplementary.pdf]

nutrients MDPI

Supplementary Materials

Table S1. Indexers used to select publications.

**PubMed 878 + 11 updated = 889**	("Carbon Footprint"[MeSH Terms] OR "Carbon Footprints"[All Fields] OR "footprint carbon"[All Fields] OR "Water"[MeSH Terms] OR "Hydrogen Oxide"[All Fields] OR "Water Resources"[MeSH Terms] OR "resource water"[All Fields] OR "resources water"[All Fields] OR "Water Resource"[All Fields] OR "environmental indicators"[MeSH Terms] OR "Environmental Indicator"[All Fields] OR "indicator environmental"[All Fields] OR "indicators environmental"[All Fields] OR "Environmental Impacts"[All Fields] OR "Environmental Impact"[All Fields]) AND ("Meals"[MeSH Terms] OR "Meal"[All Fields] OR "Mealtimes"[All Fields] OR "Meal Times"[All Fields] OR "Meal Time"[All Fields] OR "Mealtime"[All Fields] OR "Dinner Time"[All Fields] OR "Dinner Times"[All Fields] OR "Dinnertime"[All Fields] OR "Dinnertimes"[All Fields] OR "Dinner"[All Fields] OR "Dinners"[All Fields] OR "Supper"[All Fields] OR "Suppers"[All Fields] OR "Menu Planning"[MeSH Terms] OR "food services"[MeSH Terms] OR "Food Service"[All Fields] OR "Meals on Wheels"[All Fields])
**Embase 1074**	#1 'carbon footprint'/exp OR 'carbon footprint'/syn<br>#2 'water resources'/exp OR 'water resources'/syn<br>#3 'environmental indicators'/exp OR 'environmental indicators'/syn<br>#4 'meals'/exp OR 'meals'/syn<br>#5 'food services'/exp OR 'food services'/syn #<br>#1 OR #2 OR #3 AND #4 OR #5<br>**#8**<br>#4 AND #7<br>1,074<br>• Edit<br>• Email alert<br>• RSS feed<br>**#7**<br>#5 OR #6<br>170,762<br>**#6**<br>**'food services'**/exp OR **'food services'**/syn<br>37,793<br>**#5**<br>**'meal'**/exp OR **'meal'**/syn<br>137,462<br>**#4**<br>#1 OR #2 OR #3<br>108,548<br>**#3**<br>**'environmental indicator'**/exp OR **'environmental indica-tors'**<br>661<br>**#2**<br>**'water resources'**/exp OR **'water resources'**/syn<br>95,536<br>**#1**

	**'carbon footprint'**/exp OR **'carbon footprint'**/syn
**LILACS (by BVS) 314**	("Carbon Footprint" OR "Pegada de Carbono" OR " Huella de Carbono" OR "Empreinte carbone" OR "Water" OR "Água" OR "Agua" OR "Eau" OR "Water Resources" OR "Recursos Hídricos" OR "Ressources en eau" OR "environmental indicators" OR "Indicadores Ambientais" OR "Indicadores Ambientales" OR "Indicateurs environnementaux") AND ("Meals" OR "Refeições" OR "Comidas" OR "Rapas" OR "food services" OR "Serviços de Alimentação" OR " Servicios de Alimentación" OR "Services alimentaires") AND (db:("LILACS"))
**Other databases**:"IBECS"AND "BINACIS **(by BVS) : 105**	("Carbon Footprint" OR "Pegada de Carbono" OR " Huella de Carbono" OR "Empreinte carbone" OR "Water" OR "Água" OR "Agua" OR "Eau" OR "Water Resources" OR "Recursos Hídricos" OR "Ressources en eau" OR "environmental indicators" OR "Indicadores Ambientais" OR "Indicadores Ambientales" OR "Indicateurs environnementaux") AND ("Meals" OR "Refeições" OR "Comidas" OR "Rapas" OR "food services" OR "Serviços de Alimentação" OR " Servicios de Alimentación" OR "Services alimentaires") AND (db:"IBECS" OR "BINACIS"))
**Web of Science 74**	(carbon footprint) OR (water resources) OR (environmental indicators)<br>AND<br>(meal) OR (food services)
**Scopus 186**	"carbon footprint" OR "water resources" OR "environmental indicators"<br>AND<br>"meal" OR "food services"

Table S2. Full-text excluded articles and reasons.

Author (year)	Reference	Exclusion Reason
Aziz et al., 2022 *	[1]	2
Aytekin-Sahin et al., 2023 *	[2]	1
Baldwin et al., 2010 *	[3]	2
Batlle-Bayer et al., 2021	[4]	3
Borges et al., 2019 *	[5]	2
Byggmästar, 2019 *	[6]	1
Falciano et al., 2022 *	[7]	2
Ferraz et al., 2020	[8]	5
Garzillo et al., 2019	[9]	4
Jin et al., 2021	[10]	2
Justo et al., 2022 *	[11]	1
Kiehle et al., 2023	[12]	1
Long et al., 2023	[13]	3
Malan et al., 2022 *	[14]	1
Mistretta et al., 2019	[15]	1
Pang et al., 2023	[16]	3
Razali et al., 2023 *	[17]	2
Rico et al., 2019 *	[18]	1
Rivera and Azapagic, 2019	[19]	2
Saxe et al., 2017	[20]	2
Saxe et al., 2018	[21]	2
Schwarz and Bonhotal, 2018 *	[22]	1

Sha'ari et al., 2023	[23]	1
Sherry and Tivona, 2022 *	[24]	2
Silva et al., 2023 *	[25]	1
Soregaroli et al., 2021 *	[26]	1
Strasburg et al., 2021 *	[27]	1
Subramanian et al., 2021 *	[28]	2
Thiel et al., 2021	[29]	1
Tonini et al., 2018	[30]	1
Torres et al., 2021 *	[31]	3
Virtanen et al., 2010 *	[32]	1
Woolley et al., 2020	[33]	3
Zamri et al., 2022 *	[34]	2
Özgen et al., 2021 *	[35]	1
Garcia et al., 2019	[36]	2
Colombo et al.,2020	[37]	6
Dahmani et al., 2022	[38]	6
Kluczkovski et al.,2022	[39]	6
Wickramasinghe et al., 2017	[40]	6
Wickramasinghe et al., 2016	[41]	6

Legend – Exclusion criteria: 1 - Resources that don't include food menu (n= 16); 2 - No assessment of the environmental resources of interest (n= 12); 3 - Short follow up time (n= 5); 4 - Guideline or manuals (n= 1); 5 - Systematic review (n=1); 6- GHGE*Excluded studies for hand search.

References

1. Aziz, N.S.A.A.; Hasmady, N.I.I.; Shafie, F.A.; Yatim, S.R.M.; Azmi, A.; Clark, A. Food Waste and Carbon Footprint Assessment of Eateries in Kelantan, Malaysia. *Malaysian Journal of Medicine and Health Sciences* **2022**, *18*, 1–8, doi:https://doi.org/10.47836/mjmhs.18.s15.1.
2. Aytekin-Sahin, G.; Besparmak, A.; Sagir, S.S.; Somtas, A.; Ozturk, D. Relationship between Nutrient Profiles, Carbon Footprint and Water Footprint of Hospital Menus. *Nutrition & Food Science* **2023**, *54*, 319–333, doi:https://doi.org/10.1108/nfs-07-2023-0154.
3. Baldwin, C.; Wilberforce, N.; Kapur, A. Restaurant and Food Service Life Cycle Assessment and Development of a Sustainability Standard. *The International Journal of Life Cycle Assessment* **2010**, *16*, 40–49, doi:https://doi.org/10.1007/s11367-010-0234-x.
4. Batlle-Bayer, L.; Bala, A.; Aldaco, R.; Vidal-Monés, B.; Colomé, R.; Fullana-i-Palmer, P. An Explorative Assessment of Environmental and Nutritional Benefits of Introducing Low-Carbon Meals to Barcelona Schools. *Science of the Total Environment* **2021**, *756*, 143879, doi:https://doi.org/10.1016/j.scitotenv.2020.143879.
5. Borges, M.P.; Souza, L.H.R.; Pinho, S. de; Pinho, L. de Impacto de Uma Campanha Para Redução de Desperdício de Alimentos Em Um Restaurante Universitário. *Engenharia Sanitaria E Ambiental* **2019**, *24*, 843–848, doi:https://doi.org/10.1590/s1413-41522019187411.
6. Byggmästar, A. *Carbon Footprint Calculation in the Restaurant Sector*; University of Vaasa : School of Technology and Innovation Industrial Systems Analytics , 2019;
7. Falciano, A.; Cimini, A.; Masi, P.; Moresi, M. Carbon Footprint of a Typical Neapolitan Pizzeria. *Sustainability* **2022**, *14*, 3125, doi:https://doi.org/10.3390/su14053125.
8. Ferraz, A.S.; Gonçalo, C.; Serra, D.; Carvalhosa, F.; Real, H. Água: A Pegada Hídrica No Setor Alimentar E as Potenciais Consequências Futuras. *Acta Portuguesa De Nutrição* **2020**, *22*, doi:https://doi.org/10.21011/apn.2020.2208.
9. Garzillo, J.M.F.; Machado, P.P.; Louzada, M.L. da C.; Levy, R.B.; Monteiro, C.A. *Pegadas Dos Alimentos E Das Preparações Culinárias Consumidos No Brasil*; Universidade De São Paulo. Faculdade De Saúde Pública, 2019; ISBN 9788588848368.
10. Jin, W.; Zhi, G.; Zhang, Y.; Wang, L.; Guo, S.; Zhang, Y.; Xue, Z.; Zhang, X.; Du, J.; Zhang, H.; et al. Toward a National Emission Inventory for the Catering Industry in China. *Science of the Total Environment* **2021**, *754*, 142184, doi:https://doi.org/10.1016/j.scitotenv.2020.142184.
11. Justo, A. de S.; Teruel, B.J.; Junior, H.G. Estimativa Da Pegada Hídrica No Campus Da UNICAMP. *International Workshop for Innovation in Safe Drinking Water* **2022**, doi:https://doi.org/10.20396/iwisdw.n1.2022.4803.
12. Kiehle, J.; Kopsakangas-Savolainen, M.; Hilli, M.; Pongrácz, E. Carbon Footprint at Institutions of Higher Education: The Case of the University of Oulu. *Journal of Environmental Management* **2023**, *329*, 117056, doi:https://doi.org/10.1016/j.jenvman.2022.117056.
13. Long, Y.; Huang, L.; Fujie, R.; He, P.; Chen, Z.; Xu, X.; Yoshida, Y. Carbon Footprint and Embodied Nutrition Evaluation of 388 Recipes. *Scientific Data* **2023**, *10*, 794, doi:https://doi.org/10.1038/s41597-023-02702-1.

14. Malan, H.; Bartolotto, C.; Wilcots, C.; Angelis, P.; Ferrone, A.; Fabris, E.; Wible, C.; Westbrook, E.; Wang, M.C.; Slusser, W.; et al. Increasing the Selection of Low-Carbon-Footprint Entrées through the Addition of New Menu Items and a Social Marketing Campaign in University Dining. *Journal of the Association for Consumer Research* **2022**, *7*, doi:https://doi.org/10.1086/720450.
15. Mistretta, M.; Caputo, P.; Cellura, M.; Cusenza, M.A. Energy and Environmental Life Cycle Assessment of an Institutional Catering Service: An Italian Case Study. *Science of the Total Environment* **2019**, *657*, 1150–1160, doi:https://doi.org/10.1016/j.scitotenv.2018.12.131.
16. Pang, M.; Zhang, Q.; Zhou, J.; Yin, Q.; Tan, Q.; Zhong, X.; Zhang, H.; Zhao, L.; Yang, Y.; Huang, Y.; et al. Dietary Patterns and Environmental Impacts of Chongqing Hotpot in China. *Resources Conservation and Recycling* **2023**, *198*, 107118–107118, doi:https://doi.org/10.1016/j.resconrec.2023.107118.
17. Razali, N.A.; Kamaruddin, N.H.; Zulfakar, A.; Kamaludin, N.H.; Alwi, N.; Shafie, F.A. Food Waste and Carbon Footprint Assessment on Selected Food Service Establishments on the East Coast of Malaysia. *Journal of Sustainability Science and management/Journal of Sustainability Science and Management* **2023**, *18*, 58–67, doi:https://doi.org/10.46754/jssm.2023.11.004.
18. Rico, A.; Martínez-Blanco, J.; Montlleó, M.; Rodríguez, G.; Tavares, N.; Arias, A.; Oliver-Solà, J. Carbon Footprint of Tourism in Barcelona. *Tourism Management* **2019**, *70*, 491–504, doi:https://doi.org/10.1016/j.tourman.2018.09.012.
19. Rivera, X.C.S.; Azapagic, A. Life Cycle Environmental Impacts of Ready-Made Meals Considering Different Cuisines and Recipes. *Science of the Total Environment* **2019**, *660*, 1168–1181, doi:https://doi.org/10.1016/j.scitotenv.2019.01.069.
20. Saxe, H.; Loftager Okkels, S.; Jensen, J. How to Obtain Forty Percent Less Environmental Impact by Healthy, Protein-Optimized Snacks for Older Adults. *International Journal of Environmental Research and Public Health* **2017**, *14*, 1514, doi:https://doi.org/10.3390/ijerph14121514.
21. Saxe, H.; Jensen, J.D.; Bølling Laugesen, S.M.; Bredie, W.L.P. Environmental Impact of Meal Service Catering for Dependent Senior Citizens in Danish Municipalities. *The International Journal of Life Cycle Assessment* **2018**, *24*, 654–666, doi:https://doi.org/10.1007/s11367-018-1487-z.
22. Schwarz, M.; Bonhotal, J. Carbon Footprint of a University Compost Facility: Case Study of Cornell Farm Services. *Compost Science & Utilization* **2018**, *26*, 128–143, doi:https://doi.org/10.1080/1065657x.2018.1438934.
23. Sha'ari, N.S.M.; Sazali, U.S.; Zolkipli, A.T.; Vargas, R.Q.; Shafie, F.A. Environmental Assessment of Casual Dining Restaurants in Urban and Suburban Areas of Peninsular Malaysia during the COVID-19 Pandemic. *Environmental Monitoring and Assessment* **2023**, *195*, doi:https://doi.org/10.1007/s10661-023-10937-z.
24. Sherry, J.; Tivona, S. Reducing the Environmental Impact of Food Service in Universities Using Life Cycle Assessment. *International Journal of Sustainability in Higher Education* **2022**, *23*, doi:https://doi.org/10.1108/ijshe-06-2021-0224.
25. Silva, T.T.C.D.; Falco, B.B.; de Castro, I.G.; Zanon, R.B.; Guerra, J.V.V.; Yaginuma, K.Y.; Oliveira, V.; Oliveira, A.G. de M. de Carbon, Water, Ecological Footprints, Energy and Nutritional Densities of Omnivore and Vegan Culinary Preparations. *Food and Nutrition Sciences* **2023**, *14*, 626–637, doi:https://doi.org/10.4236/fns.2023.147041.
26. Soregaroli, C.; Ricci, E.C.; Stranieri, S.; Nayga, R.M.; Capri, E.; Castellari, E. Carbon Footprint Information, Prices, and Restaurant Wine Choices by Customers: A Natural Field Experiment. *Ecological Economics* **2021**, *186*, 107061, doi:https://doi.org/10.1016/j.ecolecon.2021.107061.
27. Strasburg, V.J.; Fontoura, L.S.; Bennedetti, L.V.; Camargo, E.P.L.; Sousa, B.J. de; Seabra, L.M.J. Environmental Impacts of the Water Footprint and Waste Generation from Inputs Used in the Meals of Workers in a Brazilian Public Hospital. *Research, Society and Development* **2021**, *10*, e22510313129, doi:https://doi.org/10.33448/rsd-v10i3.13129.
28. Subramanian, K.; Chopra, S.S.; Wharton, C.M.; Yonge, W.; Allen, J.; Stevens, R.; Fahy, S.; Milindi, P.S. Mapping the Food Waste-Energy-Water-Emissions Nexus at Commercial Kitchens: A Systems Approach for a More Sustainable Food Service Sector. *Journal of Cleaner Production* **2021**, *301*, 126856, doi:https://doi.org/10.1016/j.jclepro.2021.126856.
29. Thiel, C.L.; Park, S.; Musicus, A.A.; Agins, J.; Gan, J.; Held, J.; Horrocks, A.; Bragg, M.A. Waste Generation and Carbon Emissions of a Hospital Kitchen in the US: Potential for Waste Diversion and Carbon Reductions. *PLOS ONE* **2021**, *16*, e0247616, doi:https://doi.org/10.1371/journal.pone.0247616.
30. Tonini, D.; Albizzati, P.F.; Astrup, T.F. Environmental Impacts of Food Waste: Learnings and Challenges from a Case Study on UK. *Waste Management* **2018**, *76*, 744–766, doi:https://doi.org/10.1016/j.wasman.2018.03.032.
31. Torres, M.L.; Maynard, D. da C. *Pegada Hídrica E Pegada De Carbono: Uma Análise De Cardápio De UAN Escolar*; Congresso Internacional Em Produção De Refeições, Alimentação E Nutrição, 2021;
32. Virtanen, Y.; Kurppa, S.; Saarinen, M.; Mäenpää, I.; Mäkelä, J.; Grönroos, J. *Carbon Footprint of Food – an Approach from National Level and from a Food Portion*; 9th European IFSA Symposium: Vienna (Austria), 2010;
33. Woolley, K.; Bartington, S.E.; Pope, F.D.; Price, M.J.; Thomas, G.N.; Kabera, T. Biomass Cooking Carbon Monoxide Levels in Commercial Canteens in Kigali, Rwanda. *Archives of Environmental & Occupational Health* **2020**, *76*, 75–85, doi:https://doi.org/10.1080/19338244.2020.1761279.
34. Zamri , N.Z.M.; Ayob, S.N.F.; Ahmad, N.A.; Noor, M.A.M.; Shafie, F.A. Environmental Assessment on Daily Operation of Selected Food Service Establishment in Northern Region of Malaysia*. *Journal of Sustainability Science and management/Journal of Sustainability Science and Management* **2022**, *17*, 67–77, doi:https://doi.org/10.46754/jssm.2022.12.007.
35. Özgen, I.; Binboğa, G.; Güneş, S.T. An Assessment of the Carbon Footprint of Restaurants Based on Energy Consumption: A Case Study of a Local Pizza Chain in Turkey. *Journal of Foodservice Business Research* **2021**, *24*, 709–729, doi:https://doi.org/10.1080/15378020.2021.1889910.

36. García-Herrero, L.; De Menna, F.; Vittuari, M. Food Waste At School. The Environmental And Cost Impact Of A Canteen Meal. Waste Management 2019, 100, 249–258, Doi:Https://Doi.Org/10.1016/J.Wasman.2019.09.027.
37. Colombo, P.E.; Patterson, E.; Lindroos, A.K.; Parlesak, A.; Elinder, L.S. Sustainable And Acceptable School Meals Through Optimization Analysis: An Intervention Study. Nutrition Journal 2020, 19, 1–15, Doi:Https://Doi.Org/10.1186/S12937-020-00579-Z.
38. Dahmani, J.; Nicklaus, S.; Grenier, J.-M.; Marty, L. Nutritional Quality And Greenhouse Gas Emissions Of Vegetarian And Non-Vegetarian Primary School Meals: A Case Study In Dijon, France. Frontiers In Nutrition 2022, 9, Doi:Https://Doi.Org/10.3389/Fnut.2022.997144.
39. Kluczkovski, A.; Menezes, C.A.; Da Silva, J.T.; Bastos, L.; Lait, R.; Cook, J.; Cruz, B.; Cerqueira, B.; Lago, R.M.R.S.; Gomes, A.N.; Et Al. An Environmental And Nutritional Evaluation Of School Food Menus In Bahia, Brazil That Contribute To Local Public Policy To Promote Sustainability. Nutrients 2022, 14, 1519, Doi:Https://Doi.Org/10.3390/Nu14071519.
40. Wickramasinghe, K.K.; Rayner, M.; Goldacre, M.; Townsend, N.; Scarborough, P. Contribution Of Healthy And Unhealthy Primary School Meals To Greenhouse Gas Emissions In England: Linking Nutritional Data And Greenhouse Gas Emission Data Of Diets. European Journal Of Clinical Nutrition 2016, 70, 1162–1167, Doi:Https://Doi.Org/10.1038/Ejcn.2016.101.
41. Wickramasinghe, K.; Rayner, M.; Goldacre, M.; Townsend, N.; Scarborough, P. Environmental And Nutrition Impact Of Achieving New School Food Plan Recommendations In The Primary School Meals Sector In England. Bmj Open 2017, 7, E013840, Doi:Https://Doi.Org/10.1136/Bmjopen-2016-013840.
